# Higher DNA methylation of ABO gene promoter is associated with acute myocardial infarction in a hospital-based population in Karachi

**DOI:** 10.12669/pjms.36.3.1406

**Published:** 2020

**Authors:** Farzana Abubakar Yousuf, Khawar Kazmi, Junaid Iqbal, Nikhat Ahmed, Mohammad Perwaiz Iqbal

**Affiliations:** 1 Farzana Abubakar Yousuf, Department of Biological and Biomedical Sciences, Aga Khan University, Karachi 74800, Pakistan; 2 Khawar Kazmi, National Institute of Cardiovascular Diseases, Karachi, Pakistan; 3 Junaid Iqbal, Department of Biological and Biomedical Sciences, Aga Khan University, Karachi 74800, Pakistan; 4 Nikhat Ahmed, Department of Biochemistry, University of Karachi, Karachi, Pakistan; 5 Mohammad Perwaiz Iqbal, Pakistan Academy of Sciences, Islamabad - Pakistan. Department of Biological and Biomedical Sciences, Aga Khan University, Karachi 74800, Pakistan

**Keywords:** ABO gene promoter, Acute myocardial infarction, Coronary artery disease, DNA methylation, Pakistani population

## Abstract

**Objective:**

To find out if there is any relationship of methylation status of ABO gene promoter with the risk of acute myocardial infarction (AMI) in a hospital-based Pakistani population in Karachi, Pakistan.

**Methods:**

A case control study comprising of 39 adult AMI patients (both males and females; age range 30-70 years) and 39 normal healthy controls (both males and females and similar age range) nested in a large study (to see the relationship of ABO genotypes with AMI) was designed to investigate the methylation status of ABO gene promoter and its association with AMI. The study was carried out at the Aga Khan University, Karachi during July 2018 to June 2019. DNA isolated from samples of AMI patients and normal healthy controls were converted into bisulphite DNA using a kit method. Methylation specific polymerase chain reaction was carried out to determine the methylation status of ABO gene promoter in both cases and controls. Logistic regression was used to find out any association between increased methylation status of ABO gene promoter and risk of AMI.

**Results:**

A significantly higher percentage of DNA methylation of the ABO gene promoter was observed in AMI patients as compared to normal healthy controls (82.1% vs. 35.9%; p value <0.001). This higher methylation status of ABO gene promoter was associated with AMI and the odds of AMI in this population were more than 6-fold in subjects with methylated gene promoter compared to those with unmethylated gene promoter after adjusting with age and waist circumference [AOR (95% CI) = 6.27 (1.76-22.3); p value = 0.005].

**Conclusion:**

The ABO gene promoter’s hypermethylation appears to be increasing the risk of AMI in a hospital-based Pakistani population in Karachi, Pakistan.

## INTRODUCTION

ABO gene locus encodes for glycosyl transferases which modify the terminal oligosaccharides of the precursor H antigen, thereby making antigen A and antigen B. While A and B alleles of ABO gene are enzymatically active, the O allele is inactive, and therefore cannot modify precursor H antigen leading to O antigen.^1^ Therefore, expression of ABO gene locus is associated with the expression of ABO phenotypes in individuals.

A systematic review by Chen *et al.*, 2016 has shown an association of blood group A and non O blood groups with increased risk of coronary artery disease (CAD).^2^ A recent report from China indicated that blood group A was an independent risk factor for severity of CAD.^3^ These reports show that expression of ABO gene has an indirect relationship with the development of CAD.

DNA methylation is an epigenetic modulation mechanism in which cytosine bases of the eukaryotic DNA immediately followed by guanine residues i.e. CpG (cytosine-phosphate-guanine) islands present in the promoter region are converted to 5 methylcytosine with the help of the enzyme DNA methyltransferases.^4^ ABO gene promoter region has a CpG island whose methylation status correlated with gene expression in the cell lines.^5^ A study by Zaina *et al*. showed an association of differentially methylated CpG islands with the onset of atherosclerosis and endothelial dysfunction.^6^ Since ABO gene locus has also been identified to be associated with myocardial infarction in patients with CAD symptoms,^7^ it is imperative to investigate the methylation status of ABO gene promoter to see whether it has a relationship with acute myocardial infarction (AMI) in a Pakistani population which has one of the highest known rates of CAD in the world.^8^ While a few studies have been carried out to study the effect of epigenetic factors on some of the clinical diseases such as metabolic diseases and psychiatric illnesses among South Asian immigrants in the West, hardly any investigation on the role of DNA methylation and the development of premature CAD has been conducted on South Asians.^9^,^10^ Therefore the objective of the study was to find out if there is any relationship of methylation status of ABO gene promoter with the risk of AMI in a hospital-based Pakistani population.

## METHODS

### Study design, sampling and sample size

This is a case control study nested in a large cohort study which had the purpose to investigate the relationship of ABO genotypes with AMI in a Pakistani population in Karachi. For the large study, the AMI patients’ samples (both males and females with the age range from 30-70 years and having a confirmed diagnosis of AMI) had been obtained from the National Institute of Cardiovascular Disease (NICVD), Karachi with informed consent, while normal healthy subjects (both males and females with same age range from 30-70 years and not suffering from any chronic disease including CAD) were recruited from the personnel of the Aga Khan University (AKU) and NICVD. The study had the approval of the Ethics Review Committees (ERC) (Ref#: 3048-BBS-ERC-14 dated on July 17, 2018) of the AKU and NICVD (Ref#: ERC-10/2015 dated on May 30, 2015). The nested current case control study was carried out at the AKU during the period July 2018 to June 2019. DNA samples isolated from AMI patients (n=39) and normal healthy controls (n=39) were randomly selected from the samples collected for the large study.

### Sample Size:

In a case control design with power of at least 80% and level of significance of 5%, a sample size of 39 in each patients group (cases) and healthy subjects group (control) was worked out when exposure ranged from 10% - 45% with an anticipated odds ratio of four or more.^11^ Therefore, 39 DNA samples of AMI patients and 39 DNA samples of healthy controls were employed for methylation profiling of ABO gene promoter to investigate any association of methylation status of promoter and risk of AMI in this population. Demographic and clinical characteristics of patients and healthy controls such as gender, age, body mass index (BMI), waist circumference (WC) and blood pressure were obtained from the record of the study participants.

### DNA Extraction and Bisulfite Conversion

Salting out method was used for isolation of genomic DNA from white blood cells of AMI patients and normal healthy control subjects using standard protocol.^12^ The amount of DNA was quantitated at 260 nm and the purity was checked by taking the optical density (OD) at the ratio 260/280. The DNA methylation experiments were then carried out on the purified DNA.

The MethylEasy™ Xceed Rapid DNA Bisulphite Modification Kit (Genetic signatures company, Australia) was used for the conversion of DNA into bisulphite DNA following manufacturer’s instruction. In this process, cytosine is converted to uracil but does not convert 5-methylcytosine (5-mc) bases as they are unreactive. The genomic DNA (5 µg) obtained from both the study groups (cases and controls) along with a positive control 1 (untreated DNA, as provided in the kit) was used. The integrity of the DNA samples after bisulphite conversion was checked by nested PCR using primers 3A (first round) and 3B (second round) and a control sample 2 (which contained bisulphite treated DNA) as positive control (all provided in the kit). The product size of the amplicon was 240bp that was analyzed on a 2% agarose gel. Now the bisulfite treated DNA samples were ready to be used in methylation specific PCR (MSP) for the profiling of ABO gene promoter.

### Methylation specific PCR (MSP)

The MSP technique detects the average DNA methylation status of any gene. The targeted methylated region of the human ABO gene was amplified by using methylation specific primers and unmethylated specific primers as described by Kominato *et al*.^5^ The unmethylated primer sequences for forward and reverse primers were (U1) 5’GGATAGGGTTTTAAGGTATTAGGGTTATG3’ and (U2) 5’CCACATCTAATCTCAACCTCCA3’, respectively. The steps for the amplifications of unmethylated PCR reaction were: initial denaturation for nine minutes at 95°C (one cycle only), then 38 repeated cycles of one minute at 94°C, one minute at 60°C, two minute at 72°C and extension at 72°C for 10 minutes. The methylation specific primer sequences of forward and reverse primers were M1 5’TTAAGGTATTAGGGTTACGAGGGGC3’ and M2 5’CGACCATAACTCCGCGTCT3’, respectively. The amplification of the methylated PCR reaction was performed as described in the following: Initial denaturation at 95°C for 9 minutes (one cycle only) and then 38 repeated cycles at 94°C for one minute, 67°C 1 minute, 72°C two minutes and extension at 72°C for 10 minutes. The total reaction volume was 50 µl containing two units of AmpliTaq Gold® 360 DNA polymerase (Applied Biosystems®) and 20 ng of bisulfite converted DNA. The 2% agarose gel containing ethidium bromide was used to visualize the amplified PCR products which had a size of 280bp.

### Statistical Analysis

Demographic and clinical data were analyzed using Statistical Package for Social Sciences (SPSS) software version 19 for Windows (Apache Software Foundation, USA) by IBM. The mean ± SD values of continuous variables (age, BMI, WC, blood pressure) between cases and controls were analyzed by Independent samples t test. The frequencies of methylated and unmethylated ABO gene promoter among AMI patients and normal healthy controls were compared using Chi-Square test. In order to study the association of methylation status of ABO gene promoter, binary logistic regression was used. A p value < 0.05 was considered statistically significant.

## RESULTS

**Fig.1:**
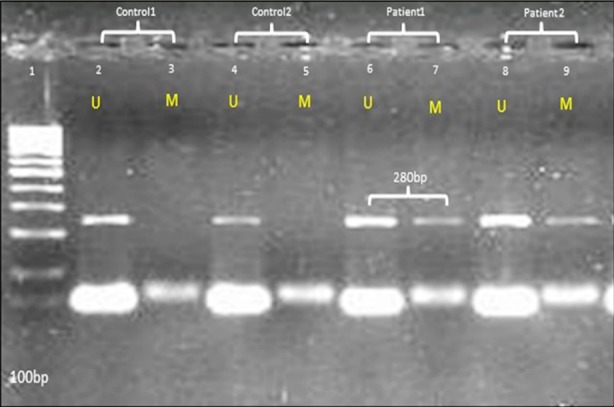
Methylation specific PCR (MSP) amplified products using methylated primer set marked as M and unmethylated primer ser marked as U for ABO gene promoter in AMI patients and normal and normal healthy controls. Lane 1= 100bp Marker, Lane 2-5= Normal healthy control samples (band in only unmethylated), Lane 6-9= AMI patients samples (bands in both unmethylated and methylated), M = Methylated PCR product, U = Unmethylated PCR product.

Fig.1 shows the gel picture of a typical methylation specific PCR (MSP). Being a sensitive technique it enables the examination of all CpG sites. Moreover, it permits simultaneous detection of both methylated and unmethylated products in a single DNA sample.^13^

The baseline demographic and clinical characteristics of both AMI patients and normal healthy subjects serving as controls have been compared and indicate statistically significant differences between the two groups in age and waist circumference (p value < 0.001 and p value = 0.005, respectively), while no significant differences were observed with respect to gender, BMI, systolic blood pressure and diastolic blood pressure (Table-I). Frequency distribution of methylated and unmethylated ABO gene promoter in Pakistani AMI patients and normal healthy controls has been shown Table-II. Significantly higher proportion of DNA methylation has been found in the ABO gene promoter of AMI patients as compared to healthy controls (82.1% vs. 35.9%; p value < 0.001). Binary logistic regression analysis revealed an association of higher methylation status of ABO gene promoter with AMI and the odds of AMI were 6.27 folds higher in methylated ABO gene promoter subjects compared to subjects with unmethylated ABO gene promoter, when adjusted for age and waist circumference [AOR (95% CI) = 6.27 (1.76-22.3); p value 0.005, Table-III]. As epigenetic changes, in general, increase with age,^14^ the regression model to study association of promoter with AMI was adjusted for age. Moreover, waist circumference has been reported to be a better predictor of CAD compared to BMI in Pakistani population,^15^ therefore, the logistic regression model was also adjusted for waist circumference. When the ABO gene promoter was hypermethylated, the odds of having AMI increased more than 6 folds in this population.

**Table-I:** Baseline and clinical characteristics of normal healthy controls and patients with acute myocardial infarction (AMI) in a Pakistani population.

Characteristics	Normal healthy controls (n =39) n (%)	AMI patients (n =39) n (%)	p value *
Gender			
Males	32(82.1)	33(84.6)	0.99
Females	7 (17.9)	6 (15.4)	

	*Mean ± SD*	*Mean ± SD*	

Age (years)	42.13 ± 7.68	53.87±8.87	**< 0.001**
BMI (kg/m^2^)	25.1 ± 3.8	25.8 ± 3.8	0.473
WC (cm)	90.3 ± 8.7	96.4 ± 9.8	**0.005**
SBP (mm of Hg)	119 ± 14	120 ± 14	0.75
DBP (mm of Hg)	78 ± 7	78 ± 12	0.95

BMI: body mass index, WC: waist circumference,

SBP: systolic blood pressure, DBP: diastolic blood pressure.

* p value represents the comparison of percentages in two groups using chi-square, while means were compared using Independent samples t test. A p value < 0.05 was considered as significant.

**Table-II:** Frequency distribution of methylated and unmethylated status of ABO gene promoter in a Pakistani population.

	Status of ABO gene promoter		

Subjects	Unmethylated (%)	Methylated (%)	P value*
Cases (n=39)	7 (17.9)	32 (82.1)	< 0.001
Controls (n=39)	25 (64.1)	14 (35.9)	

* p value was determined using chi-square test. p value < 0.05 was considered as significant.

**Table-III:** Association of ABO gene promoter’s methylation with the risk of AMI in a Pakistani population using binary logistic regression.

Status of ABO gene promoter	Crude OR (95% CI)	p value	Adjusted OR (AOR)^1^ (95% CI)	p value
Unmethylated	1.0		1.0	
Methylated	8.1 (2.86-23.26)	<0.001	6.27 (1.76-22.3)	0.005

1 Adjusted for age and waist circumference.

## DISCUSSION

Coronary heart disease (CHD) is the major cause of death in Pakistan.^8^ Another study from Pakistan indicated that prevalence of CAD was more than 6% in people of the age greater than 30 years.^16^ Since non-O group individuals predominate in this population, it is conceivable that the risk of CAD would be high in this population. The underlying mechanism of association of non-O blood group antigens with CAD has not been defined clearly, however there are reports which show that non-O group subjects have 25% higher levels of factor VIII-vWF complex compared to group O individuals and vWF (von Willibrand factor) is known to have a role in thrombosis by mediating the adhesion of platelets to vascular wall and promoting their aggregation, thereby increasing the risk of CAD.^17^ Moreover, expression of ABO gene locus is also associated with plasma levels of lipids. For example, blood group A subjects have been found to have higher levels of total cholesterol and low-density lipoprotein cholesterol.^18^ Another possible mechanism for the association of blood groups A and non-O could be increased expression of inflammatory molecules such as tumor necrosis factor alpha which is known to mediate endothelial cell activation by increasing the expression of various adhesion molecules.^17^ All these reports show the possible mechanism linking ABO gene locus with the risk of CAD. However, most of these lines of evidence deal with traditional risk factors of CHD. Besides these traditional risk factors, epigenetic factors could also be playing some role for the high prevalence of this disease in Pakistan. Epigenetic refers to changes in gene expression without alteration in DNA sequence in response to environmental factors such as smoking, diet and lack of physical activity.^19^ DNA methylation on the CpG islands in the gene promoter region is one of the most studied epigenetic modifications.^20^ It has been shown to regulate the expression of genes associated with cardiovascular disease (CVD) risk factors including atherosclerosis, inflammation, hypertension and diabetes mellitus.^21^,^22^ Recent studies have shown ABO gene locus as a susceptibility locus for CAD, MI and other diseases, and the environmental factors and epigenetic signatures contribute in the process of development of CAD.^23^ There is evidence of association of non-O blood groups to the risk of developing CAD.^2^ However, in certain populations, there was no significant association of non-O group with the risk of CAD.^24^ This indicates that this association could vary from one population to another.

There has been no report in the literature describing the relationship of expression of ABO gene promoter with the risk of CAD in South Asian population. Kominato *et al*. had described that hypermethylation of ABO gene promoter caused silencing of the gene in stomach carcinoma cell line.^5^ Later Bianco-Miotto *et al.*, reported that hypermethylation of ABO promoter was associated with the loss of ABO allelic expression in patients with leukemia.^25^ Therefore, the current study is unique in reporting the association of higher methylation of ABO gene promoter to the risk of AMI in a Pakistani population. The mechanism by which hypermethylation of ABO gene promoter could be related to the risk of AMI is unclear. However, it can be conjectured that similar to the allelic loss observed in patients with hematological malignancies,^25^ there could be a loss of ABH antigens in patients with AMI with associated complications such as thrombosis and altered endothelial function.^26^ Duygu *et al.*, have reported association of DNA methylation with arrhythmias and heart failure.^27^ Though they have not described the mechanism involved, but their findings do indicate a relationship of gene silencing with heart disease.

### Limitations of the study

Though the sample size was modest, however, the study had adequate power to investigate the relationship between hypermethylation of promoter and risk of AMI. Furthermore, the design of the study was cross sectional; therefore, it could only provide information about association or relationship between hypermethylation of ABO gene promoter and risk of AMI. No cause-effect relationship could be inferred from such a design. In order to obtain information whether hypermethylation could be the cause of AMI, some kind of longitudinal studies would be required. Another limitation of the current study is the lack of detailed information about various drugs and medications used by the patients and healthy controls because certain drugs have been shown to alter the epigenetic profiling to a favorable status.^28^ Thus, large studies involving measurements at different time intervals along with complete information about medicines used, their diet, physical activity and comorbids would be required to obtain some conclusive evidence regarding the role of epigenetics in causing CAD in Pakistani population.

## CONCLUSION

There is an association between higher methylation of ABO gene promoter and the risk of AMI in a hospital-based Pakistani population. Since the epigenetic changes are reversible, they offer exciting opportunities for better treatment and management of chronic diseases such as CVD.

### Authors Contribution:

**FAY, KK, JI, NA** **&** **MPI** conceived the experiments,

**FAY, JI & MPI:** were involved in data collection and statistical analysis and drafted the manuscript. They are responsible for the accuracy or integrity of the work.

**JI, KK, MPI & NA** reviewed the manuscript.
